# Release of elements and phenolic and flavonoid compounds from herbs and spices into acacia honey during infusion

**DOI:** 10.1007/s13197-024-06019-8

**Published:** 2024-06-17

**Authors:** Nikolett Czipa, Clive J. C. Phillips, Emőke Topa, Béla Kovács

**Affiliations:** 1 Institute of Food Science, Faculty of Agricultural and Food Sciences and Environmental Management, University of Debrecen, Böszörményi Street 138, Debrecen, 4032 Hungary; 2 Sustainable Policy Institute, Curtin University, Perth, WA Australia

**Keywords:** Honey, Infusion, Spice/herb, Element content, Total phenolic content, Flavonoid content

## Abstract

Acacia honey was infused with basil, oregano, marjoram, dill, garlic or cinnamon at infusion rates of 0–5% by mass for a 6 months period. After removal of the infusates, macro and micro element concentrations were measured by Inductively Coupled Plasma Optical Emission Spectrometry. Total phenolic and flavonoid contents were determined spectroscopically. The greatest release of elements, phenols and flavonoids, (% release/1% infusion rate) were for phenols (1.22–3.74, respectively), flavonoids (0.12–2.18), K (0.39–0.78), P (0.14–0.87), and S (0.07–0.85). The least release was for Ba (0.04–0.17), Fe (0.03–0.41) and B (− 0.006 to 2.33). Dill showed the most important effect on the Na concentration of honey enriched (at 5.00%) with > 90 times higher content (328 ± 4 mg/kg) compared to control honey (3.46 ± 0.07 mg/kg). Sr content was more than 50 times higher in honey enriched with marjoram (1383 ± 10 µg/kg), and honey enriched with dill showed more than 30 times higher Fe content (4112 ± 14 µg/kg). Enrichment with dill had the greatest effect on Ca, Cu, K, Mg, Na and Fe content of control honey, and garlic had the most important effect on the B, P, S, Zn, TP and TF content. Enrichment with these herbs and spices resulted in increases in element, total phenolic and flavonoid content of acacia honey.

## Introduction

Honey is a naturally sweet food produced by *Apis mellifera* honeybees, originating from nectar or honeydew. In Hungary the most important type is from the acacia tree, which grows well in this geographical and climatic location. Honey has long been recognised for its health-giving properties, mostly resulting from secondary metabolites from the plants transmitted to the honey by the bees (Sowa et al. 2019).

Spices have therapeutic effects due to their bioactive components, therefore these medicinal plants have widely used in traditional treatment of different diseases. Most herbs are used against gastrointestinal disorders (e.g. diarrhoea and vomiting) due to their antibacterial and antiviral properties (El-Saber Batiha et al. 2020). Basil, oregano and cinnamon can be used in the treatment of inflammation of the urinary track; basil, oregano and garlic help in the treatment of asthma (Ahmed et al. 2019; Morshedloo et al. 2018; Thomas and Duethi 2001). Oregano and cinnamon can be used in the case of menstrual disorders (Morshedloo et al. 2018; Thomas and Duethi 2001); basil, garlic and dill can help in the treatment of cancer (Ahmed et al. 2019; El-Saber Batiha et al. 2020; Carvalho et al. 2016). Garlic can reduce blood pressure and blood lipids and it has benefits in the treatment of diabetes and inflammation (Myneni et al. 2016). Cinnamon is used in the treatment of liver problems (Thomas and Duethi 2001).

Recently, attempts have been made to combine health giving properties of honeys with those of herbs and spices, by infusing the latter in the honey and removing it before sale. Due to the unique chemical composition of honey, it can serve an excellent medium for herbal infusion. Infused honeys with different herbs have therapeutic effect and these products may be use in treatment of gastrointestinal and respiratory disorders, dermatological issues and metabolic syndromes (Kumar et al. 2024).

There has been little scientific study of honey that has been enriched with different spices/herbs; with only articles published that have focused on their physicochemical and antioxidant properties. Štajner et al. (2014) examined the physicochemical properties (e.g. TPC, vitamin C, flavonoid content, pH) of honey enriched with *rosa spp*. Wilczyńska et al. (2017) determined the taste, aroma, texture, colour and appearance of honey enriched with cardamom, cinnamon and ginger. Dżugan et al. (2017) observed creamed multi-floral honey enriched with lavender, lemon balm, nettle, peppermint and ginger and measured their antioxidant properties. Ng et al. (2023) examined the sensory, physicochemical and antioxidant properties of honey enriched with ginger extract. Content of rutin and quercetin, as well as the sensory properties of acacia honeys enriched with Sophora flower were analysed by Đorđević et al. (2022). According to Abderrahim et al. (2019) evidence is beginning to emerge that a combination of garlic and honey may have synergistic effects on the treatment of pathogenic bacteria in humans.

However, as well as transferring bioactive compounds into the honey, infusing herbs and spices also potentially enriches the elemental content of the honey. The mineral content of honey is very low, about 0.1–0.3% in floral honey (Rodríguez-Ramos et al. 2020). Phenol contents in honey are not high, and flavonoid contents are very low, especially in acacia honeys compared to other honey types (Becerril-Sánchez et al. 2021). Based on our previous studies, acacia honey has the lowest element, total phenolic and flavonoid contents of all honey types (e.g. linden, chestnut, forest, sunflower, rape) (Czipa et al. 2010, 2015, 2018). To the best of our knowledge, no-one has examined the element concentration of honey enriched infused with spices or herbs. The aims of this study were to determine the effect of enrichment with different spices/herbs on the element content, total phenolic and flavonoid contents of acacia honey.

## Material and methods

### Sample preparation

The base honey for the experiment was from acacia (*Robinia pseudoacacia*), collected directly from a Hungarian beekeeper in sterile glass jar in 2018, then stored in the dark at room temperature until being mixed with spices and herbs. Dried and ground spices/herbs (cinnamon from Indonesia, basil from Egypt, oregano from Turkey, marjoram from Egypt, dill from Hungary and garlic from Slovakia) were obtained from a local supermarket in 2018. These samples were stored in the dark at room temperature in their original packaging. Honey samples (100 g) were prepared with 0 (Control), 1.25, 2.50, 3.75 and 5.00% (by weight) addition of these spices/herbs and then stored in the dark at room temperature for a 6 months infusion, during which time they were stirred every 2 weeks. After 6 months, the herbs and spice were removed from the samples.

### Analytical methods

All chemicals were analytical grade or better. Ultrapure water (18.2 MΩ) was used to prepare the solutions and dilutions produced by Milli-Q water purification system (Millipore S.A.S., Molsheim, France).

#### Determination of element content

The digestion of samples for element analysis was carried out using the method of Czipa et al. (2018), which is based on the methodology of Kovács et al. (1996), validated for both plant and animal materials. Applied reagents were nitric acid (69% v/v; VWR International Ltd., Radnor, USA) and hydrogen-peroxide (30% v/v; VWR International Ltd., Radnor, USA). After preparation of the solution and filtration (Sartorius Stedim Biotech S.A., Gottingen, Germany), the determination of element content was carried out using ICP-OES (Thermo Scientific iCAP 6300, Cambridge, UK). The element standard solutions were prepared from mono-elemental standard solutions (1000 mg/L; Scharlab S.L., Barcelona, Spain). Operating parameters for the determination were as follows: operating power 1150 W, plasma gas flow rate 12 L/min, auxiliary gas flow rate 1.0 L/min, nebulizer gas flow rate 1.0 L/min, rinsing time 35 s, stabilization time 3.0 s, integration time 20 and 10 s (for axial and radial WL range). Software for ICP-OES (iTEVA) was used to determine limits of detection (LOD) in blank reagent samples (*n* = 10). LODs (mg/kg) of ICP-OES were: 0.012 for B, 0.009 for Ba, 0.134 for Ca, 0.037 for Cu, 0.055 for Fe, 0.675 for K, 0.137 for Mg, 0.012 for Mn, 0.015 for Na, 0.325 for P, 0.239 for S, 0.011 for Sr and 0.063 for Zn.

#### Determination of total phenolic content (TPC) and total flavonoid content (TFC)

TPC was determined by the Folin-Ciocalteu method (Singleton et al. 1999), using a spectrophotometer (Evolution 300 LC, Thermo Electron Corporation, England) at 760 nm. Applied reagents were: Folin-Ciocalteu reagent (0.2 N; VWR International S.A.S., France), sodium-carbonate (75 g/L; Scharlab S.L., Spain), methanol:distillate water solution (80:20; Scharlab S.L., Spain), and Gallic acid (Alfa Aesar GmbH & Co. KG, Karlsruhe, Germany). TPC was expressed in mg gallic acid equivalent (GAE)/100 g.

TFC was determined according to the method of Kim et al. (2003), using a spectrophotometer (Evolution 300 LC, Thermo Electron Corporation, England) at 510 nm. Applied reagents were: sodium-nitrite (VWR International, S.A.S., France), aluminium-chloride (VWR International, S.A.S., France), sodium-hydroxide (Sigma-Aldrich Chemie GmbH, Steinheim, Germany), methanol:distillate water solution (80:20; Scharlab S.L., Spain), catechin (VWR International, S.A.S., France). TFC was expressed in mg catechin equivalent (CE)/100 g.

### Statistical analysis

**Fig. 1:**
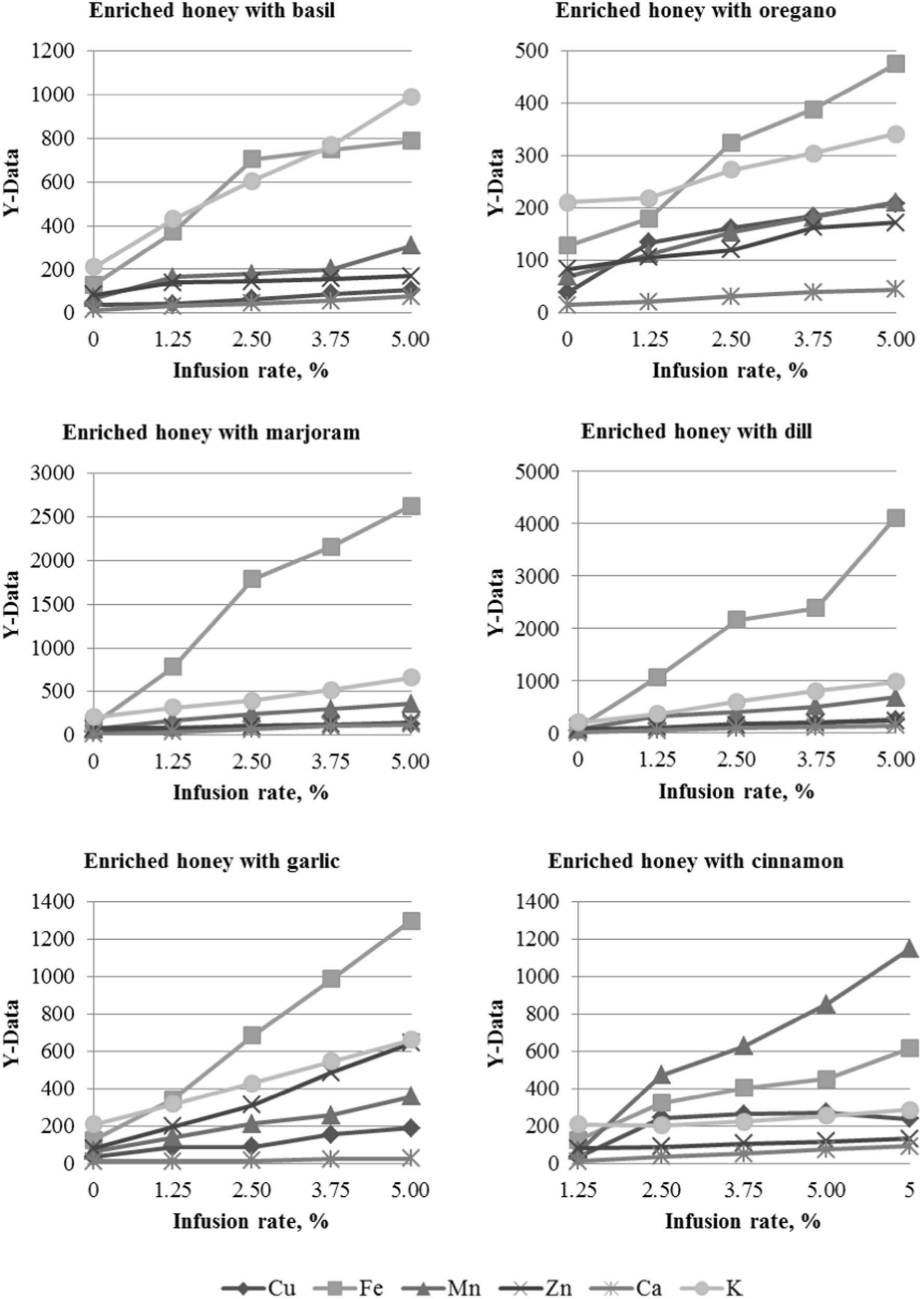
Scatterplot of Cu, Fe, Mn, Zn, Ca and K vs Infusion rate, %

All analytical determinations were carried out in triplicate. Data were described using standard descriptive statistics (mean and standard deviation) and One-way ANOVA with Dunnett’s T3 test (significance was assumed at *P* < 0.05). Analyses were checked for homogeneity of variances. SPSS for Windows (version 13; SPSS Inc. Chicago, Illinois, USA) was utilised for these initial calculations. Then the proportional element releases were calculated by linear regression of concentrations of each element against the inclusion rate of the herb/spice, since there was no evidence of non-linear release into the honey (Fig. 1). This used the equation y = mx + c, where y = element concentration in honey and x = herb/spice inclusion rate. The m values were divided by the original concentration in the herb/spice and multiplied by 100 to get the release of each element and TPC and TFC, in % of original concentration in the herb/spice. Two one-way analyses of variance were used to compare, first, the release of the different elements across herbs/spices and, second, the release rate between different herbs/spices. Differences between individual elements or herbs/spices were examined by Fisher’s Exact Test. These analyses utilised the software package Minitab.

## Results

### Macro element content of honey enriched

Table 1 shows the macro element content of honey enriched. Basil had the highest Ca content; however, the honey enriched with basil showed lower Ca concentrations than those enriched with dill, marjoram and cinnamon. Similar Ca concentrations were observed in marjoram and dill, and honey enriched with these herbs (5.00% inclusion rate) had similar concentrations (*P* value = 0.44). Enrichment with garlic at 1.25 and 2.50% did not show a significant increase in Ca, compared with the control honey (*P* values = 0.16 and 0.07, respectively). However, nine and eight times higher Ca contents were detected in honey enriched with marjoram and dill at 5.00%, compared to control honey. This value was only two times higher in the case of honey enriched with garlic.

**Table 1:** Macroelement concentrations of honey enriched with basil, oregano, marjoram, dill, garlic and cinnamon included at concentrations of 1.25–5%

Sample	Spice/herb addition, %	Ca (mg/kg)	K (mg/kg)	Mg (mg/kg)	Na (mg/kg)	P (mg/kg)	S (mg/kg)
*Control honey*	*–*	*15.4* ± *0.3*	*211* ± *4*	*3.69* ± *0.11*	*3.46* ± *0.07*	*35.9* ± *0.7*	*13.9* ± *0.5*
**Basil**	*–*	**20,030 ± 94**	**30,141 ± 661**	**6788 ± 20**	**454 ± 5**	**3884 ± 19**	**2632 ± 53**
Honey enriched with basil	1.25	32.3 ± 0.8	430 ± 7	12.9 ± 0.4	7.69 ± 0.16	44.0 ± 0.3	18.8 ± 0.1
	2.50	45.7 ± 0.8	604 ± 10	19.4 ± 0.5	10.9 ± 0.4	49.4 ± 0.7	21.9 ± 0.2
	3.75	57.9 ± 0.3	768 ± 3	26.7 ± 0.8	15.7 ± 0.6	56.0 ± 0.3	25.3 ± 0.3
	5.00	77.5 ± 0.6	992 ± 4	34.7 ± 0.4	18.5 ± 0.3	63.5 ± 0.6	30.9 ± 0.2
**Oregano**	*–*	**13,292 ± 321**	**6683 ± 16**	**1968 ± 61**	**39.1 ± 1.6**	**1091 ± 7**	**1477 ± 9**
Honey enriched with oregano	1.25	22.1 ± 0.3	219 ± 3	5.82 ± 0.06	2.60 ± 0.06	37.6 ± 0.1	16.4 ± 0.4
	2.50	31.6 ± 0.6	273 ± 5	8.62 ± 0.18	2.64 ± 0.06	41.5 ± 0.6	18.9 ± 0.4
	3.75	39.7 ± 0.5	305 ± 5	10.2 ± 0.1	3.12 ± 0.06	44.3 ± 0.7	21.5 ± 0.1
	5.00	45.2 ± 1.0	342 ± 3	12.3 ± 0.5	4.39 ± 0.02	48.2 ± 0.9	23.9 ± 0.6
**Marjoram**	**–**	**17,361 ± 40**	**15,491 ± 363**	**3815 ± 113**	**800 ± 19**	**2500 ± 81**	**3959 ± 6**
Honey enriched with marjoram	1.25	37.1 ± 0.7	317 ± 3	10.1 ± 0.4	7.43 ± 0.05	38.5 ± 0.4	26.5 ± 0.6
	2.50	69.6 ± 0.6	399 ± 2	14.2 ± 0.2	13.3 ± 0.2	51.7 ± 0.7	38.1 ± 0.8
	3.75	108 ± 1	519 ± 7	21.5 ± 0.3	18.9 ± 0.2	60.0 ± 0.9	52.6 ± 0.2
	5.00	126 ± 6	659 ± 9	26.7 ± 0.3	23.1 ± 0.9	66.5 ± 0.4	75.6 ± 0.5
**Dill**	**–**	**16,158 ± 198**	**24,570 ± 223**	**3729 ± 114**	**11,109 ± 94**	**3268 ± 40**	**7318 ± 43**
Honey enriched with dill	1.25	52.1 ± 1.3	367 ± 3	16.4 ± 0.3	94.0 ± 0.8	54.0 ± 1.4	49.5 ± 1.7
	2.50	89.3 ± 1.8	608 ± 5	27.8 ± 0.7	185 ± 5	68.3 ± 0.5	69.4 ± 1.0
	3.75	117 ± 4	806 ± 9	36.3 ± 1.0	252 ± 4	78.4 ± 1.5	89.6 ± 2.2
	5.00	140 ± 3	977 ± 4	45.6 ± 0.1	328 ± 4	87.3 ± 1.0	113 ± 1
**Garlic**	**–**	**1264 ± 7**	**11,603 ± 134**	**795 ± 4**	**462 ± 3**	**3703 ± 65**	**5014 ± 64**
Honey enriched with garlic	1.25	13.9 ± 0.4	319 ± 3	9.52 ± 0.08	7.19 ± 0.07	77.2 ± 0.8	68.0 ± 0.5
	2.50	18.7 ± 0.6	432 ± 2	15.4 ± 0.3	10.3 ± 0.2	116 ± 2	122 ± 2
	3.75	25.3 ± 0.6	550 ± 3	22.8 ± 0.4	13.7 ± 0.1	160 ± 2	181 ± 5
	5.00	29.2 ± 0.6	664 ± 10	28.8 ± 0.4	17.8 ± 0.2	197 ± 1	226 ± 4
**Cinnamon**	**–**	**10,418 ± 278**	**4018 ± 71**	**1033 ± 24**	**37.4 ± 0.3**	**533 ± 3**	**1383 ± 43**
Honey enriched with cinnamon	1.25	41.5 ± 1.1	206 ± 5	4.25 ± 0.06	2.02 ± 0.03	39.0 ± 0.5	16.5 ± 0.5
	2.50	54.6 ± 0.7	227 ± 4	4.81 ± 0.08	2.21 ± 0.02	40.8 ± 0.4	15.8 ± 0.4
	3.75	76.9 ± 0.4	257 ± 3	5.28 ± 0.04	2.32 ± 0.03	43.6 ± 0.6	17.5 ± 0.4
	5.00	94.7 ± 1.9	289 ± 2	6.10 ± 0.06	2.42 ± 0.04	46.6 ± 0.9	18.5 ± 0.6

Italics value indicates the results of honey

Bold value indicates the results of spices

The highest K content was determined in basil, followed by dill, marjoram, garlic, oregano and cinnamon. At 2.50, 3.75 and 5.00% honey enriched with basil and dill did not show statistically significant differences in K content (*P* values = 1.00, 0.14 and 0.27, respectively). There were no statistically significant differences between honey enriched with marjoram and garlic at 1.25, 3.75 and 5.00% infusion rates (*P* values = 1.00, 0.14, 1.00, respectively). Enrichment with oregano (1.25%) and cinnamon (1.25 and 2.50%) did not show any significant difference from control honey (*P* values = 0.62, 0.99 and 0.15, respectively). More than four times higher K contents were observed in honey enriched enriched with basil and dill compared to control honey. This value was less than twice in the case of honey enriched enriched with cinnamon and oregano.

Basil showed the highest Mg content; however honey enriched with basil showed only the second highest concentrations of all infusion rates. The highest Mg content was observed with dill even though the dill had the third highest Mg concentration. The lowest Mg content was measured in garlic followed by cinnamon and oregano; however honey enriched enriched with cinnamon had the lowest Mg concentration followed by oregano and marjoram at all infusion rates. In spite of having the lowest Mg content the honey enriched with garlic significantly increased the concentration. Marjoram and dill showed similar Mg content; however the enrichment did not result in similar concentrations. Enrichment with cinnamon at 1.25% was not significantly different in Mg concentration from control honey (*P* value = 0.09). More than 12 times and nine times higher Mg contents were measured in honey enriched with dill and basil (5.00% infusion rate); however this value was less than twice in the case of honey enriched with cinnamon.

The highest Na content was determined in dill, followed by marjoram, garlic, basil, oregano and cinnamon. The order of Na concentration in honey enriched with these herbs was corresponded to the order in spices/herbs. Cinnamon and oregano contained Na in higher content than control honey; however the addition of this spice and herb did not increase the Na content in honey enriched. Similar Na contents were determined in basil and garlic, as well as in oregano and cinnamon. There were no significant differences in Na concentrations between honey enriched with basil and garlic at all infusion rates. Due to the very high Na content of dill, a > 90 times higher Na concentration was measured in honey enriched with dill at 5.00%. In the case of other honey-enriched samples this value was much lower (between 1 and 6).

The highest *P* concentrations were measured in basil and garlic followed by dill, although the highest *P* concentrations were determined in honey enriched with garlic and dill. No significant differences between the *P* content of basil and garlic were observed, but garlic increased the *P* content more than the addition of basil. With oregano and cinnamon no significant differences in *P* concentration were observed at any infusion rates (*P* values = 0.28, 0.98, 1.00 and 0.95, respectively). Honey enriched with basil and marjoram did not show statistically significant differences in *P* concentration at 2.50, 3.75 and 5.00% infusion rates (*P* values = 0.33, 0.15 and 0.15, respectively). There were no differences between the honey enriched with cinnamon and oregano. Enrichment with oregano, marjoram and cinnamon at a rate of 1.25% did not show significant differences from control honey (*P* values = 0.40, 0.15 and 0.09, respectively). More than five times higher *P* concentration was measured in honey enriched with garlic at 5.00%. In the case of other honey-enriched samples this value was lower (between 1.3 and 2.4).

The highest S concentration was determined in dill followed by garlic, marjoram, basil, oregano and cinnamon. The order of S concentration in honey enriched with these herbs corresponded to the order in spices/herbs, except garlic for which the enrichment with garlic showed higher S contents. Enrichment with oregano (1.25%) and cinnamon (1.25 and 2.50%) did not result in significant differences from control honey (*P* values = 0.070, 0.09 and 0.16, respectively). More than 16 times higher S content was measured in honey enriched with garlic (at 5.00%).

### Micro element content of enriched honeys

Table 2 shows the micro element content of enriched honeys. The highest B content was measured in marjoram, followed by basil and dill, and the lowest in cinnamon and garlic. The enrichment at different rates resulted in similar B contents in the case of honey enriched with basil, dill, garlic and cinnamon. An increase in B was observed only in honey enriched with oregano and marjoram (with the addition of higher amounts of the herbs). The very high B content of basil, oregano, marjoram and dill did not increase the concentration of B more than a low content of garlic and cinnamon.

**Table 2:** Microelement concentrations of honey enriched with basil, oregano, marjoram, dill, garlic and cinnamon included at concentrations of 1.25–5.00%

Sample	Spice/herb addition, %	B (µg/kg)	Ba (µg/kg)	Cu (µg/kg)	Fe (µg/kg)	Mn (µg/kg)	Sr (µg/kg)	Zn (µg/kg)
*Control honey*	*–*	*475* ± *5*	*18.5* ± *1.0*	*39.1* ± *0.4*	*129* ± *6*	*69.7* ± *1.7*	*24.6* ± *0.6*	*83.7* ± *1.4*
**Basil**	**–**	**22,800 ± 30**	**27,466 ± 57**	**20,030 ± 94**	**433,963 ± 7336**	**69,662 ± 162**	**153,384 ± 2798**	**39,302 ± 752**
Honey enriched with basil	1.25	478 ± 2	26.3 ± 0.2	42.9 ± 0.7	372 ± 9	164 ± 3	184 ± 2	140 ± 6
	2.50	480 ± 4	45.6 ± 0.4	64.6 ± 1.2	706 ± 6	178 ± 2	275 ± 5	146 ± 5
	3.75	468 ± 4	64.6 ± 0.8	89.4 ± 1.2	750 ± 6	201 ± 5	386 ± 8	156 ± 5
	5.00	468 ± 1	74.2 ± 0.5	108 ± 5	789 ± 7	308 ± 3	521 ± 3	170 ± 6
**Oregano**	**–**	**20,200 ± 80**	**14,577 ± 254**	**13,292 ± 321**	**210,013 ± 5277**	**38,788 ± 162**	**37,975 ± 547**	**14,242 ± 85**
Honey enriched with oregano	1.25	466 ± 3	22.2 ± 0.4	135 ± 6	181 ± 2	112 ± 4	42.7 ± 0.4	106 ± 5
	2.50	468 ± 3	25.9 ± 0.6	162 ± 6	325 ± 5	154 ± 6	58.1 ± 0.6	121 ± 5
	3.75	526 ± 5	42.1 ± 0.7	185 ± 6	389 ± 4	183 ± 4	76.5 ± 1.4	163 ± 6
	5.00	532 ± 6	56.2 ± 0.6	209 ± 5	475 ± 12	211 ± 5	87.8 ± 0.5	172 ± 2
**Marjoram**	**–**	**29,800 ± 30**	**28,316 ± 145**	**17,361 ± 40**	**1,678,685 ± 24,120**	**69,396 ± 782**	**221,957 ± 4588**	**30,203 ± 391**
Honey enriched with marjoram	1.25	447 ± 4	30.9 ± 0.9	69.1 ± 0.2	783 ± 7	174 ± 5	480 ± 8	88.6 ± 4.9
	2.50	480 ± 4	47.6 ± 1.2	106 ± 7	1785 ± 29	244 ± 4	797 ± 3	107 ± 5
	3.75	489 ± 3	72.0 ± 0.6	115 ± 3	2162 ± 6	300 ± 1	1280 ± 8	123 ± 2
	5.00	533 ± 6	93.6 ± 2.8	123 ± 4	2627 ± 14	361 ± 7	1383 ± 10	150 ± 2
**Dill**	**–**	**22,800 ± 50**	**11,799 ± 558**	**16,158 ± 198**	**982,122 ± 9066**	**73,866 ± 842**	**98,598 ± 794**	**31,166 ± 336**
Honey enriched with dill	1.25	447 ± 5	26.3 ± 0.4	99.6 ± 0.3	1070 ± 13	325 ± 9	159 ± 7	104 ± 3
	2.50	470 ± 3	28.4 ± 0.4	182 ± 10	2168 ± 53	396 ± 9	285 ± 2	157 ± 3
	3.75	492 ± 5	37.3 ± 0.1	198 ± 6	3291 ± 35	514 ± 11	368 ± 4	196 ± 2
	5.00	498 ± 6	46.0 ± 1.0	259 ± 3	4112 ± 14	688 ± 10	443 ± 11	241 ± 5
**Garlic**	**–**	**566 ± 16**	**5358 ± 33**	**1264 ± 7**	**57,213 ± 874**	**9769 ± 142**	**13,193 ± 66**	**16,794 ± 232**
Honey enriched with garlic	1.25	533 ± 6	24.9 ± 0.1	89.1 ± 0.3	346 ± 5	142 ± 3	41.1 ± 1.9	199 ± 3
	2.50	535 ± 5	24.8 ± 0.6	91.6 ± 3.1	687 ± 12	214 ± 3	61.0 ± 0.4	314 ± 5
	3.75	515 ± 11	37.8 ± 0.3	158 ± 9	989 ± 10	260 ± 8	94.5 ± 2.3	489 ± 4
	5.00	541 ± 2	65.1 ± 2.0	193 ± 2	1299 ± 12	361 ± 6	121 ± 2	646 ± 6
**Cinnamon**	**–**	**835 ± 13**	**89,651 ± 950**	**10,418 ± 278**	**218,764 ± 5973**	**307,570 ± 4245**	**39,501 ± 310**	**11,311 ± 336**
Honey enriched with cinnamon	1.25	488 ± 1	107 ± 4	245 ± 6	325 ± 7	475 ± 9	165 ± 4	92.0 ± 0.9
	2.50	496 ± 2	167 ± 5	266 ± 6	405 ± 7	629 ± 9	230 ± 5	110 ± 1
	3.75	507 ± 7	213 ± 5	275 ± 5	454 ± 7	855 ± 12	299 ± 7	120 ± 3
	5.00	512 ± 4	284 ± 7	241 ± 5	616 ± 8	1149 ± 18	399 ± 1	135 ± 5

Italics value indicates the results of honey

Bold value indicates the results of spices

Cinnamon showed the highest Ba content followed by marjoram and basil; and the order was the same in the case of honey enriched with these spices/herbs. The lowest Ba concentration was in garlic, followed by dill and oregano; however, honey enriched with garlic had higher Ba content compared to the other two honey enriched samples. A similar content was measured in basil and marjoram, and honey enriched with these herbs did not show differences at 1.25 and 2.50% infusion rates (*P* values = 0.12 and 0.78, respectively). Oregano and dill also showed similar concentrations, and the enriched honey did not show significant differences at 2.50 and 3.75% infusion rates (*P* value = 0.14 and 0.07, respectively). Compared to control honey, the enrichment with oregano or garlic did not result in significant differences at 1.25% infusion rate (*P* values = 0.21 and 0.08). The measured Ba content was 15 times higher in honey enriched with cinnamon (5.00% infusion rate) compared to control honey; and the lowest value was 2.5 times higher.

The highest Cu content was measured in basil and marjoram; however honey enriched with these herbs had the lowest concentrations at all infusion rates. The migration of this element was the highest in the case of dill and cinnamon. Although marjoram and dill had similar Cu concentrations (*P* value = 0.08), significant differences were observed between honey enriched at all infusion rates. Enrichment with basil did not show differences from control honey at the 1.25% rate (*P* value = 0.994). Enrichment of honey at a 5.00% rate resulted in at least three times higher Cu content, while it was more than six times higher in honey enriched with dill and cinnamon.

Marjoram had the highest Fe concentration, followed by dill, and garlic had the lowest. In spite of its higher Fe content, honey enriched with marjoram showed lower concentrations than honey enriched with dill. Enrichment with garlic resulted in the third highest content of all honey enriched, which were uniformly higher in Fe than control honey. More than 30 times and 20 times higher Fe concentrations were observed in honeys enriched with dill and marjoram (at 5.00%), respectively; while it was six times higher at a 1.25% rate.

The highest Mn content was measured in cinnamon followed by dill, and the order of enriched honeys was the same as in the spices/herbs. Garlic had the lowest concentration; however higher contents were measured in the honey enriched with garlic than in honey enriched with marjoram and basil. There were no significant differences between basil, marjoram and dill herbs; however enrichment with dill showed higher Mn content. All enriched samples had significantly higher Mn contents compared to the control honey. More than nine times higher Mn content was observed in honey enriched with cinnamon at the 5.00% infusion rate.

Marjoram showed the highest Sr content followed by basil, dill and cinnamon, and the order was the same in the herbs/spices as the honey enriched with these spices/herbs. The lowest concentration was measured in garlic followed by oregano; however the honey enriched with garlic had higher concentrations at 3.75 and 5.00% infusion rates. There was no statistically significant difference between oregano and cinnamon; however, these enriched honeys showed significant differences at all infusion rates. All enriched honeys had significantly higher Sr contents compared to control honey. More than 50 times higher Sr content was observed in honey enriched with marjoram at 5.00%; while it was only three times in the case of honey enriched with oregano at 5.00%.

The highest Zn content was determined in basil; however the migration of this element into honey was low. Garlic had the fourth lowest Zn concentration; however, the honey enriched with garlic had the highest content of Zn. Marjoram and dill had similar Zn contents, but the enrichment with these herbs produced different Zn contents in the honey. Honey enriched with oregano, marjoram and dill showed no significant increase, compared to control honey, at 1.25% infusion rate (*P* value = 0.12, 0.97 and 0.06). More than seven times higher Zn content was measured in honey enriched with garlic at 5.00%.

### TPC and TFC of honey enriched

Table 3 shows the TPC and TFC of honey enriched. Cinnamon and marjoram had the highest TPCs followed by dill, basil, oregano and garlic; and the order was the same in case of honey enriched with these spices/herbs at 5.00% infusion rates. Honey enriched with cinnamon showed the highest TPCs followed by marjoram and dill at all infusion rates. Due to the high TPCs of cinnamon and marjoram, more than 11 times higher and seven times higher contents were measured in honey enriched at 5.00%. Basil and oregano showed similar TPCs; however, honey enriched with oregano had higher contents at 1.25, 2.50 and 3.75% infusion rates. In case of honey enriched with dill, basil and oregano three–four times higher TPCs were determined at 5.00% infusion rate. Garlic had the lowest measured TPC, and honey enriched with this herb showed the lowest values.

**Table 3:** TPC and TFC of honey enriched with basil, oregano, marjoram, dill, garlic and cinnamon included at concentrations of 1.25–5.00%

Sample	Spice/herb addition, %	TPC (mgGAE/100 g)	TFC (mgCE/100 g)	Sample	Spice/herb addition, %	TPC (mgGAE/100 g)	TFC (mgCE/100 g)
*Control honey*	*–*	*14.1* ± *0.2*	*1.94* ± *0.11*	*Control honey*	*–*	*14.1* ± *0.2*	*1.94* ± *0.11*
**Basil**	**–**	**464 ± 6**	**1884 ± 6**	**Dill**	**–**	**527 ± 10**	**1399 ± 13**
Honey enriched with basil	1.25	20.2 ± 0.1	7.56 ± 0.06	Honey enriched with dill	1.25	27.0 ± 0.4	9.23 ± 0.11
	2.50	25.9 ± 0.3	13.0 ± 0.2		2.50	41.7 ± 0.2	18.1 ± 0.4
	3.75	34.8 ± 0.6	20.3 ± 0.3		3.75	47.6 ± 0.4	24.0 ± 0.3
	5.00	42.4 ± 0.5	28.7 ± 0.4		5.00	56.9 ± 0.4	32.6 ± 0.7
**Oregano**	**–**	**451 ± 4**	**2862 ± 5**	**Garlic**	**–**	**124 ± 3**	**17.9 ± 0.2**
Honey enriched with oregano	1.25	22.4 ± 0.2	6.15 ± 0.12	Honey enriched with garlic	1.25	15.9 ± 0.3	3.07 ± 0.05
	2.50	31.5 ± 0.3	11.2 ± 0.3		2.50	18.1 ± 0.3	3.38 ± 0.08
	3.75	37.9 ± 0.2	15.9 ± 0.3		3.75	21.0 ± 0.1	3.65 ± 0.06
	5.00	41.9 ± 0.1	19.5 ± 0.4		5.00	23.1 ± 0.3	3.89 ± 0.03
**Marjoram**	**–**	**689 ± 5**	**3369 ± 9**	**Cinnamon**	**–**	**780 ± 6**	**4667 ± 28**
Honey enriched with marjoram	1.25	46.2 ± 0.3	20.1 ± 0.5	Honey enriched with cinnamon	1.25	56.7 ± 0.4	40.6 ± 0.7
	2.50	61.3 ± 0.4	40.5 ± 0.8		2.50	93.2 ± 1.0	78.4 ± 0.5
	3.75	97.2 ± 0.5	76.9 ± 0.4		3.75	119 ± 1	110 ± 3
	5.00	102 ± 2	96.0 ± 0.5		5.00	160 ± 3	177 ± 4

Italics value indicates the results of honey

Bold value indicates the results of spices

Cinnamon and marjoram had the highest TFCs, followed by oregano, basil, dill and garlic; however the order was not the same in case of honey enriched with these spices/herbs. At all infusion rates honey enriched with cinnamon had the highest contents, followed by honeys enriched with marjoram, dill, basil, oregano and garlic. In the case of cinnamon-enriched honey, a more than 90 times higher content was measured in honey enriched at the 5.00% infusion rate; and TFC was more than 20 times higher at 1.25%. Wylczinska et al. (2017) made a similar observation, 2% of cinnamon addition caused an important increase in the TPC of honey. Honey enriched with marjoram had almost 50 times higher TFC at 5.00% infusion rate. The content was 16 times, 14 times and ten times higher in honey enriched with dill, basil and oregano, respectively, at the 5.00% infusion rates. Due to the very low TFC in garlic, less than two times higher contents were measured in honey enriched at all infusion rates. Based on statistical analysis there were no significant differences among honeys enriched at 2.50, 3.75 and 5.00% infusion rates.

### Release rates of the elements, phenolic compounds and flavonoids into the honeys

There were significant differences between the Release (% release/1% infusion rate) in the various herbs and spices (SED = 0.342; F [5, 72] 7.12; *P* < 0.001). Garlic had a greater mean Release rate for all elements (0.826) than any of the spices (dill 0.227, marjoram 0.180, cinnamon 0.171, oregano 0.152 and basil 0.147). There were significant differences in the Release rates between elements (SED = 0.0938; F [12, 65] 3.17; *P* = 0.001) (Table 4). They were greater for Cu (0.586), B (0.555) and K (0.547) than all elements (Na 0.380; P 0.366; S 0.293; Mg 0.200; Zn 0.179; Mn 0.177; Ca 0.124; Sr 0.107; Fe 0.4105 and Ba 0.071).

**Table 4:** Release (% release/1% infusion rate) of each element, TPC and TFC into honey

Spice/herb	B	Ba	Ca	Cu	Fe	K	Mg	Mn
Basil	− 0.006	0.041	0.062	0.069	0.030	0.518	0.091	0.068
Oregano	0.056	0.052	0.030	0.256	0.033	0.392	0.088	0.073
Marjoram	0.039	0.053	0.127	0.097	0.030	0.578	0.121	0.084
Dill	0.020	0.047	0.154	0.272	0.081	0.624	0.225	0.167
Garlic	2.33	0.174	0.218	2.44	0.410	0.781	0.632	0.596
Cinnamon	0.889	0.059	0.152	0.388	0.045	0.388	0.047	0.070

Spice/herb	Na	P	S	Sr	Zn	TPC	TFC
Basil	0.663	0.142	0.129	0.065	0.044	1.22	0.284
Oregano	0.476	0.226	0.135	0.033	0.124	1.23	0.123
Marjoram	0.491	0.245	0.312	0.122	0.044	2.55	0.558
Dill	0.584	0.315	0.271	0.085	0.101	1.62	0.438
Garlic	0.621	0.870	0.846	0.146	0.670	1.45	2.18
Cinnamon	− 0.556	0.402	0.067	0.189	0.091	3.74	0.750

In case of TPCs cinnamon had the greatest Release, followed by marjoram, dill, garlic, oregano and basil. Examining TFCs, the greater Release rate was for garlic, followed by cinnamon, marjoram, dill, basil and oregano. TPCs showed higher Release (1.97) than TFCs (0.722).

## Discussion

Macro and micro element concentrations varied widely in the examined spices and herbs and were for all elements much greater than in honey (except B in honeys enriched with basil, oregano, marjoram and dill). The addition of herbs and spices increased element concentrations generally in proportion to the concentrations added and their concentrations in the plant. Addition at 1.25% produced an increase in element concentrations of at least three times greater than that in control honey for the following elements and herbs/spices: basil: Mg and Sr; oregano: Cu; marjoram: Fe and Sr; dill: Ca, Mg, Na, S, Fe, Mn and Sr; garlic: S; and cinnamon: Ba, Ca, Cu, Mn and Sr. At 5.00% addition, an increase in the examined element concentrations of at least five times greater than in control honey occurred as follows: basil: Ca, Mg, Na, Fe and Sr; oregano: Cu; marjoram: Ca, Mg, Na, S, Ba, Fe, Mn and Sr; dill: Ca, Mg, Na, S, Cu, Fe, Mn and Sr; garlic: Mg, Na, P, S, Fe, Mn and Zn; and cinnamon: Ca, Ba, Cu, Mn and Sr.

By comparing our results with element content of basil and oregano honeys published by other researchers, our honey enriched with basil showed higher potassium and magnesium concentrations than that determined by Jovetić et al. (2017) (201–351 mg/kg for K and 10.1–15.5 mg/kg for Mg). They measured similar calcium (44.8–49.7 mg/kg) and sodium (6.4–22.7 mg/kg) contents in basil honeys; but our honey enriched with basil (at 3.75% and 5.00%) showed higher calcium content. They had greater copper (0.20–0.380 mg/kg), iron (0.75–1.51 mg/kg), manganese (0.66–0.68 mg/kg) and zinc (1.0–3.6 mg/kg) contents in basil honeys than was measured in our honeys enriched with basil. Aazza et al. (2014) determined the macro element content of oregano honey; higher calcium (86.4 ± 3.5 mg/kg), sodium (71.5 9.4 mg/kg), potassium (623 ± 5.5 mg/kg) and magnesium (28.2 ± 1.1 mg/kg) concentrations were measured in this monofloral honey than in our honey enriched with oregano.

Control acacia honey had low TPC and very low TFC; but the addition of herbs/spices increased these contents. Enrichment with cinnamon produced the highest increase in TPC followed by marjoram, dill, oregano, basil and garlic at all infusion rates. In case of TFC the highest increase was observed with enrichment of cinnamon followed by marjoram, dill, basil, oregano and garlic at all infusion rates. However, in order of release rates for TPC cinnamon was the highest, followed by marjoram, dill, garlic, oregano and basil; and for TFC garlic was the highest, followed by cinnamon, marjoram, dill, basil and oregano.

Several studies examined the TPC and TFC contents of honey enriched with different spices and herbs. However, different methods were used, but similar conclusions may be draw from these reports to the results of our honeys enriched with various herbs and spices. Štajner et al. (2014) examined the TPC in honey infused with *Rosa* spp. and they observed an important increase (16.9 mgGAE/100 g in control honey and 155 mgGAE/100 g in honey with 10 g/100 g of *Rosa* spp). Dżugan et al. (2017) determined the TPC in creamed multifloral honeys with addition of dried lavender flower, lemon balm, nettle, peppermint leaves and ginger root (1.5% w/w); and they observed higher TPC in these honeys then was determined in herb honeys. Ng et al. (2023) measured the TPC in ginger enriched *Apis cerana* honeys and found higher TPC in these honeys (37.1 and 45.9 mgGAE/100 g) than was measured in control honey (20.2 mgGAE/100 g).

By comparing TPC and TFC of basil, oregano, marjoram and dill honeys with our honeys enriched with these herbs and spices, basil honeys examined by Sakač et al. (2022) showed higher TPC (101 ± 3 mgGAE/100 g), than our honey enriched with basil. Živković et al. (2019) measured higher TPC in oregano honey (76.2 ± 0.2 mgGAE/100 g) than was measured in our honey enriched with oregano; however higher TFC was determined in our samples at 2.50%, 3.75% and 5.00% infusion rates than in oregano honey (8.96 ± 0.17 mg CE/100 g). Attanzio et al. (2016) examined dill honeys, which showed higher TPC (95.1–133 mgGAE/100 g) and flavonoid content (75.1–82.1 mgQE/100 g) than was measured in our honey enriched with dill. However, Iranian dill honeys (Khalafi et al. 2016) showed lower flavonoid content (4.1 ± 0.2 mg/100 g) than was measured in our honey enriched with dill. They determined similar TPC (32.5 ± 1.1 mgGAE/ 100 g) to our honey enriched at 1.25%; however our samples showed higher polyphenol content at higher infusion rates.

To conclude, enrichment of acacia honey with spices/herbs significantly increased the macro and micro element concentrations as well as TPC and TFC, except B where the examined spices/herbs had no effect on B content of honey; and TPC and TFC where garlic had no important effect on these two parameters. The increase was generally in proportion to the concentration of herb or spice added to the honey.

Garlic showed the highest release of almost all examined parameters, except Sr (cinnamon), Na (basil) and TPC (cinnamon); while the lowest release rates were determined for basil (B, Ba, Cu, Mn, Zn, P and TPC), cinnamon (K, Mg, Na, S and FC), oregano (Sr and K) and marjoram (Fe).

## Conclusion

Enrichment of acacia honey with selected spices and herbs resulted in significant increase in content of macro and micronutrients. The element content, total phenolic and flavonoid content in our control acacia honey was low, however, the enriched honeys showed much higher levels than control honey. Enrichment with dill resulted in the highest increase in Ca, K, Mg, Na and Cu concentrations, enrichment with cinnamon showed the highest elevation in Ba, Cu, Mn, TPC and TFC contents, and garlic had an important effect on P, S and Zn concentrations. Garlic infused honey released the elements in garlic to a greater extent than other herbs and spices released their elements. Phenols, sodium, potassium and phosphorus were most readily released into the honey, and boron was not released to a detectable level.

Based on the results of this and other researches in relation to mixing of honey with spices, the using of any of the spices or herbs used in our study can increase the content of useful components in the honey, thereby increasing the contribution to nutritional reference intakes for minerals or antioxidant compounds.

Compared to the elements studied, total phenolic and flavonoid contents of these enriched honeys with herbs and spices suggested higher release into the honey. Based on limited data available in the literature, it is clear that there are important differences between these parameters of honeys collected from various plants, but because of limited information, it cannot be concluded that enrichment with herbs resulted in higher or lower element content or antioxidant compounds in honeys than the direct collection of nectar from these plants by honeybees. However, it can be seen that enrichment of honey with cinnamon or garlic—which are not nectar plants—resulted in some of the biggest increases in examined parameters. Therefore, research with honey enriched with cinnamon or garlic is important to investigate in future.

## Data Availability

The datasets used and analysed during the current study are available from the corresponding author on a reasonable request.

## Abbreviations

ICP-OES: Inductively coupled plasma optical emission spectrometer
TPC: Total phenolic content
TFC: Total flavonoid content
